# Designing feelings into lower-limb prostheses – A kansei engineering approach to understand lower-limb prosthetic cosmeses

**DOI:** 10.1177/20556683241289938

**Published:** 2024-10-17

**Authors:** Nerrolyn Ramstrand, Maria Riveiro, Lars Eriksson, Michael Ceder

**Affiliations:** 1 Department of Rehabilitation, School of Health and Welfare, 4161 Jönköping University, Jönköping, Sweden; 2 Department of Computer Science and Informatics, School of Engineering, 4161 Jönköping University, Jönköping, Sweden; 3 Department of Product Development, Production and Design, School of Engineering, 4161 Jönköping University, Jönköping, Sweden

**Keywords:** Affective engineering, emotional engineering, design, amputation, prosthetic limb

## Abstract

**Introduction:** This study aimed to quantify the relationship between prosthetic users’ emotional response to prosthesis aesthetics and specific product properties. **Methods:** Words representing prosthesis users’ emotional response (Kansei) to different aesthetic designs of prostheses were identified via interviews and mood boards. A group of experts consolidated the words into thematic groups, each represented by a single, high-level ‘Kansei’ word. 53 lower limb prosthesis users completed a questionnaire, rating their perception of 13 aesthetic designs using the ‘Kansei’ words. Quantification Theory Type 1 was applied to explore the relationship between words and product properties. Sub-analyses assessed for differences based on sex, age and level of extroversion. **Results:** 5 high-level Kansei words were identified (‘Natural’, ‘Technological’, ‘Cool’, ‘Unique’ and ‘Functional’). The Kansei word ‘Natural’ had a strong association with realistic looking prostheses while the words ‘Technological’, ‘Cool’ and ‘Unique’ were strongly associated with expressive designs which incorporate hard, colourful covers. The word ‘Functional’ was not a reliable predictor of product properties. No major differences were observed within sub-grouped categories. **Conclusion:** Kansei words identified in this study can be used to help guide clients in their aesthetic design choices and to assist designers in achieving the desired response from their products.

## Introduction

Products that are perceived as high quality by consumers are typically those which have the greatest affective impact, meaning that they have a positive effect on the consumer’s mood, emotions, or feelings. In the context of product development, the emotional response a person experiences from a product is referred to by the Japanese word ‘Kansei’. Kansei engineering is a product development methodology that aims to facilitate the translation of consumers’ feelings and emotions into specific design parameters.^1^ Since its introduction the Kansei method of product development has been strongly associated with consumers’ purchase choices, product use and satisfaction.^2^ While Kansei Engineering methods are used extensively to support the design of automotive and home products, examples from the medical technology industry are scarce.^3^

Satisfaction with the aesthetic design (cosmesis) of prosthetic limbs is associated with users’ level of acceptance of their prosthesis, their functional recovery and their engagement in activities that involve revealing their body.^4,5^ Despite these important findings, satisfaction with prosthetic cosmesis is often reported as low.^6^ Kansei Engineering offers a novel method by which the prosthetics industry can improve user satisfaction by facilitating the design and prescription of products and ensuring that they have a cosmetic appearance that evokes a positive emotional response from the individual user.

Vlachaki et al.^7^ have classified the cosmetic appearance of lower-limb prostheses into three distinct categories. *Realistic prostheses,* incorporating a cosmetic cover which imitates the natural appearance of a human limb, *functional prostheses* where base components of the prosthesis are exposed, and no cosmetic cover is used and *expressive prostheses* which emphasize uniqueness and visibility by using colour, texture, and finish. The category of cosmetic cover that a prosthesis user chooses is highly personal and requires that they navigate a complex interplay between their own personal preferences, cultural norms, and social perceptions. For example, realistic prostheses have been perceived as symbols of concealment, enabling users to exercise control over disclosure and allowing them the discretion to decide whether to reveal their amputation status.^8,9^ Functional prostheses have been perceived as outward manifestations of acceptance, projecting an image that the user is comfortable rejecting societal norms concerning physical appearance and has made a choice to prioritise function over aesthetics.^7^ Expressive prostheses have been perceived as a way of challenging societal notions surrounding disability.^9,10^ Hall and Orzada propose that expressive prostheses serve as vehicles for making a social statement, rejecting pressures to conform to normative body images and emphasizing uniqueness.^11^

Several attempts have been made to understand preferences regarding the cosmetic appearance of lower-limb prostheses. These generally categorise preferences on a group level and do not account for individual differences. Vlachaki et al.^12^ provided evidence to suggest that personality types impact on aesthetic design preferences, suggesting that people who are more confident prefer functional prostheses. There is also some evidence of sex related differences in aesthetic design preferences, although results vary across published studies. Vlachaki et al.^12^ reported that males in the UK preferred realistic prostheses over expressive prostheses while females reported the opposite.^12^ Murry and Fox^13^ indicated that male prosthesis users in the UK tended to prioritise functional aspects of prostheses over appearance while females prioritised appearance. In contrast, Mohad^14^ reported that male prosthesis users in Malaysia were more likely to wear a cosmetic cover than females. Contrasting results from different countries suggest that cultural norms may play an important role in aesthetic design choices. Disability related characteristics are also likely to influence preferences. For example, people who have had an amputation for a shorter period show a preference for functional prostheses while those who have had a prosthesis for a longer period prefer expressive prostheses.^12^ Amputation aetiology has also been shown to influence choices with traumatic amputees least likely to select a realistic prosthesis and people with congenital limb loss least likely to choose an expressive prosthesis.^12^ Level of amputation has not been found to influence preferences.^12^

Given that there are so many variables that can influence aesthetic design choices it is imperative that service providers understand the Kansei their clients’ desire from a new device and how to reflect and translate this into a prosthetic limb. The aim of this study was to use Kansei Engineering methods to describe the relationship between prosthetic users’ emotional response (Kansei) to lower-limb prosthetic cosmeses and product properties and to explore how personal characteristics affect the emotional response of users to aesthetic design elements of the prosthesis.

## Kansei Engineering methods

**Figure 1.**
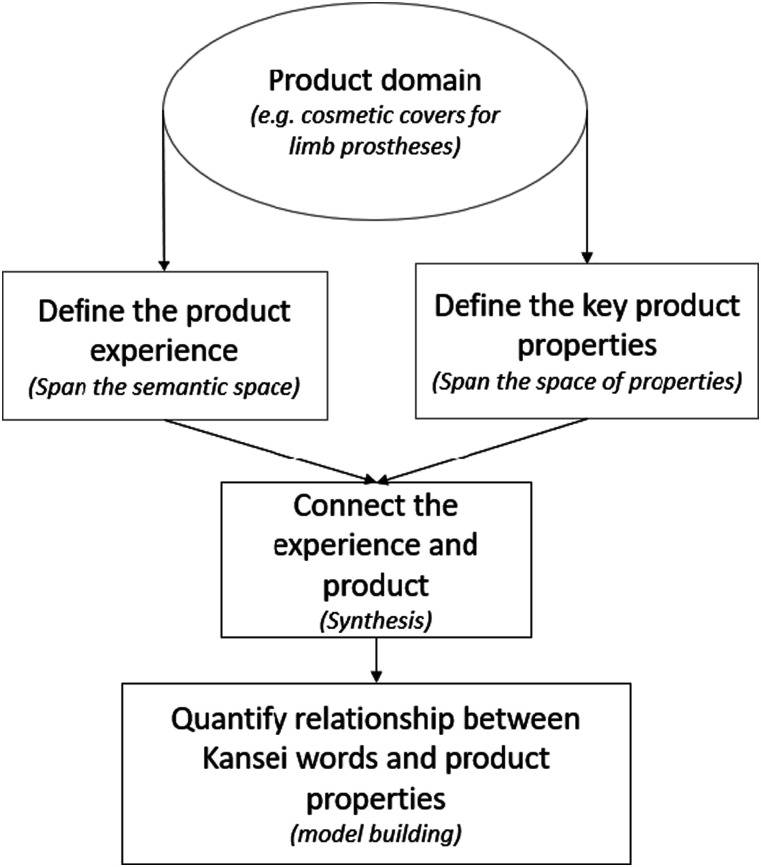
Kansei engineering model, adapted from Schütte 2005.

Kansei Engineering aims to capture a person’s psychological feelings into the design of products. This means that it is necessary to capture an affective (emotional) response to products rather than a rational (cognitive) response.^15^ Various Kansei engineering methods have been proposed as a means of incorporating human emotions, feelings, and preferences into product design and development processes.^3^ Schütte et al.^16^ propose a general model that has been applied in this study (Figure 1). Once the product domain of interest has been selected (e.g. cosmetic covers for lower-limb prostheses), Schütte proposed the following steps:

1.)** Define the product experience (Spanning the semantic space) –** An initial step in understanding the Kansei associated with specific products is to map the range of sensations that can be experienced with different product designs. This is achieved by using Kansei words, adjectives that semantically describe the product of interest. Many different techniques have been used to identify Kansei words including text searches of social media sites^17^ and product reviews,^18^ generation of mood boards^19^ and focus group discussion.^20,21^ Kansei words are hierarchic and once an extensive list has been generated researchers must condense the number of words to a manageable amount by identifying a limited number of high level words that capture the essence of multiple low-level words.^22^ For example, the low-level words ‘smooth’, ‘level’, ‘rough’ and ‘bumpy’ can be represented by one higher level word ‘finish’.
2.)** Define the key product properties (Span the space of properties) -** In this step, it is necessary to identify physical product properties that are related to the chosen product domain. These could be related to color, texture, shape, sound, smell, or any other physical trait of the product. Products typically have many properties with the potential to influence consumers’ Kansei and too many product properties are not manageable to analyse. It is subsequently necessary to limit the product properties to those perceived to have the highest emotional impact. This is most commonly done using affinity diagrams, designers’ opinions, ranking studies or Pareto diagrams.^16^
3.)** Connect the experience and product (Synthesis) -** This step requires that connections are made between Kansei words (step 1) and product properties (step 2).^23^ This is typically achieved by having a sample of individuals rate their valuation of Kanesi words for a sample of products which represent variations of the product properties defined in step 2. A common method is to have participants rate words on a visual analogue scale.^24,25^
4.)** Quantify relationship between Kansei words and product properties (Model building) –** This final step uses data collected in step 3 to quantify the relationship between Kansei words and product properties. Several tools have been proposed for this analysis including Quantification Theory Type 1 (QT1), a form of multiple linear regression,^26^ and ordinal logistic regression.^27^

## Method

The Kansei method proposed by Schütte (2005) was used to explore the link between prosthetic users’ emotional responses and specific product properties. For this project we chose to define the product domain as cosmetic covers for lower-limb prostheses. Unless otherwise stated, data was collected in Sweden and in the Swedish language. For the purposes of publication, results have been translated into English.

### Span of semantic space

An expansive list of Kansei words was generated using two different methods, 1/searching text from interview transcripts and 2/mood boards. In the first instance transcripts of individual interviews from a previously published study^28^were analysed to generate an initial list of Kansei words. Transcripts contained interviews with lower-limb prosthesis users and included open-ended questions about their experience of living with a prosthesis. Data was available from interviews with 15 Swedish prosthesis users (11 male and 4 female; 8 Trans-femoral or knee disarticulation prosthesis users and 7 trans-tibial prosthesis users). Participants were aged between 27 and 70 and original interviews ranged between 21 and 96 min. To extract data for the present study, each interview transcript was read several times by one of the authors who identified Kansei words that were used by the interviewee to describe prosthetic limbs.

To further build upon the list of Kansei words, four workshops were held to generate mood boards reflecting prostheses with different types of aesthetic design. Two workshops were held with lower-limb prosthesis users in Sweden, one group comprising of only male prosthesis users (*n* = 3) and one group comprising of only female prosthesis users (*n* = 5). One workshop was held with clinical prosthetist/orthotists in Sweden (*n* = 5) and one was held with employees from a company involved in designing and manufacturing expressive cosmetic covers for prosthetic devices in Spain (*n* = 6). This final workshop was conducted in English and Kansei words generated by the group were translated into Swedish by the first author. Participants for workshops were recruited using convenience sampling. To be eligible to participant, prosthesis users were required to be over the age of 18 and to use a lower-limb prosthesis daily. Prosthetist/orthotists were required to have experience of clinical practice in prosthetics, while designers were required to be employed within a company that develops and manufactures prosthetic products. During the workshops, participants were presented with a variety of prostheses with different cosmetic covers, including no cover, and were requested to generate a mood board reflecting each aesthetic design. Participants were instructed that mood boards should convey the feelings that they experienced with each design and were provided with a variety of magazines (e.g. lifestyle magazines, automobile magazines, fashion magazines) from which they could cut out pictures and words. When each mood board was complete participants were requested to explain and reflect on how the pictures and words that they chose reflected their feelings about the aesthetics of the prosthesis. During this reflection process, two researchers listened to the discussion and documented Kansei words used by participants to describe prosthetic devices. Words identified by each researcher were pooled and duplicates removed.

In accordance with the methods described by Schütte et al.,^16^ an expert panel was recruited to consolidate the list of Kansei words that had been generated from interview transcripts and mood boards and to identify a limited number of high-level words representative of the semantic space. The expert panel included 2 prosthesis users (1 male and 1 female), 3 clinical prosthetists (2 male and 1 female), 2 industrial designers (male) and 1 engineer (female). The consolidation meeting was held online. During the meeting participants were presented with all Kansei words on a digital whiteboard and were requested to group them into themes by moving them around with their computer mouse. During this process participants were not permitted to talk to each other and were instructed that it was permissible to move a word that someone else had already categorised. When it was clear that no additional categories were being generated, participants were encouraged to discuss the placement of words and agree on a final grouping. In the final step they were asked to identify a single high-level Kansei word to represent each category of low-level words. This final collection of high-level words was considered to define the semantic space of cosmetic covers for lower-limb prostheses.

### Space of product properties

To determine which product properties should be included in this study we recruited a group of experts in the field of prosthesis manufacture and design to participate in a focus group discussion. This group included 5 certified prosthetists, one engineer and one industrial designer. These experts were requested to collaborate to generate a comprehensive list of all attributes of cosmetic covers for lower limb prostheses that are possible to modify. They were then requested to prioritise these attributes and reach a consensus on 4 to 5 product properties that they perceived to have the greatest influence on users’ emotional response to their prosthesis.

### Synthesis

To explore the relationship between Kansei words and product properties we manufactured and photographed 13 prosthetic limbs that represented variations of the product properties identified by experts (see Table 1). This included prosthetic limbs representing each of the three different aesthetic design categories described by Vlachaki et al.^7^ (realistic prostheses, functional prostheses and expressive prostheses). Expressive prostheses included those in which the device was covered by a purpose designed prosthetic sock (prostheses 7-10) and devices that were 3D printed (prostheses 11-13). Photographs of each prosthesis were imported into Kansei Engineering Software (KESo)^29^ and a questionnaire developed in which people could rate each of the 13 prosthetic limbs. The questionnaire was designed so that respondents were presented with a picture of each limb together with visual analogue scales (VAS) that were100 mm long, allowing respondents to rate each prosthesis according to each Kansei word. VAS scales were anchored at each end with “very little” and “very much”.

**Table 1.** Prosthetic designs representing variations of the product properties selected by experts.

	Realistic prostheses				Functional prostheses		
	1	2	3	4	5	6
Form	Organic	Organic	Organic	Organic	Organic	Tech
Material	Matt	Matt	Matt	Shiny	Shiny	Shiny
Colour	Neutral	Neutral	Neutral	Neutral	Neutral	Neutral
Surface	Soft	Soft	Hard	Hard	Hard	Hard
	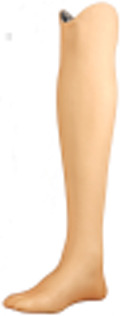	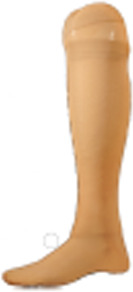	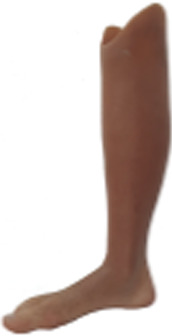	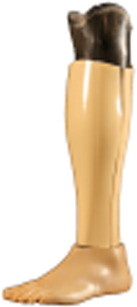	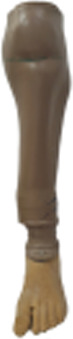	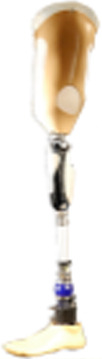
	Expressive prostheses						
	7	8	9	10	11	12	13
Form	Organic	Organic	Tech	Tech	Organic	Organic	Organic
Material	Shiny	Matt	Shiny	Matt	Matt	Shiny	Shiny
Colour	Colourful	Colourful	Colourful	Colourful	Colourful	Colourful	Colourful
Surface	Soft	Soft	Hard	Hard	Hard	Hard	Hard
	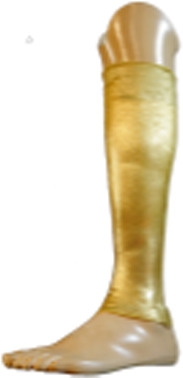	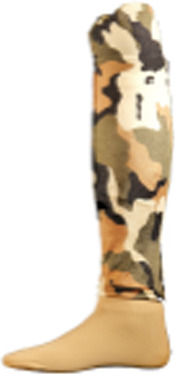	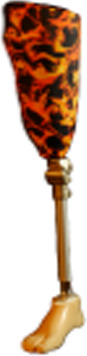	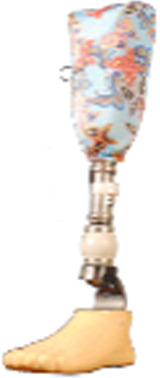	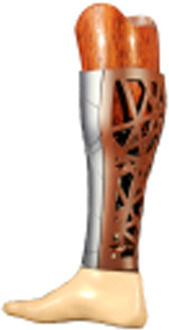	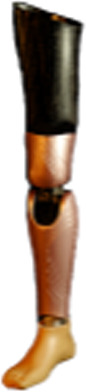	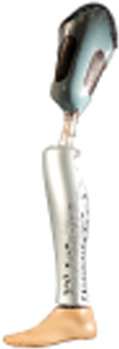

To evaluate how personal characteristics may influence an individual’s Kansei, the questionnaire also included items related to the respondents’ age, sex, time since amputation, level of amputation, satisfaction with appearance of their current prosthesis (0 = not satisfied at all; 10 = completely satisfied), reason for amputation and the type of cosmetic cover they currently used. To determine if personality influenced Kansei we included the 8 items that form the extraversion subscale of the Big 5 inventory.^30^ This subscale is validated in Swedish.^31^

A link to the questionnaire was distributed to lower-limb prosthesis users via rehabilitation clinics in Sweden and posted, with permission of administrators, on a closed Facebook group specifically for prosthesis users in Sweden. Methods were evaluated and approved by the Swedish Ethical Review Authority (ref 2023-01045-02).

### Analysis of questionnaire data

Descriptive statistics were used to summarise demographics of the sample and radar diagrams were produced to compare ratings of Kansei words for each prosthesis. Visual inspection of Q-Q plots were used to evaluate if data were normally distributed. Mean scores for each Kansei word were compared across Vlachaki et al’s categories of prostheses (realistic, functional and expressive)^7^ using a one-way repeated measures ANOVA. When significant differences were observed, paired sample t-tests with Bonferroni corrections (alpha values divided by the number of tests) were used to evaluate where differences occurred between each category of prosthesis.

To quantify the relationship between Kansei words and product properties Hayashi’s QT1^32^ was used to create a linear regression model. Goodness of fit of the model was initially assessed for each semantic word by calculating the multiple correlation coefficient (MCC). An MCC of +1 reflects perfect prediction and more reliable results while an MCC of 0 suggests no better than random prediction.^33^ An MCC above 0.6 was considered as acceptable.^23^ A partial correlation coefficient (PCC) was calculated to determine which product properties had the strongest relationship with Kansei words and a score for specific product properties was used to indicate how categories affected each word.

As previous work has indicated that aesthetic design preferences may be influenced by personality type, sex, age, and time since amputation, separate models were generated for each of these variables. To facilitate this analysis a post-hoc categorisation of variables was performed. In the absence of a strict cutoff for extroversion scores, personality types were divided into three categories defined as low extroversion (<25), moderate extroversion (26-35) and high extroversion (36+). The influence of sex was evaluated by performing separate analyses for males and females, while age was evaluated for three groups (18-45, 46-65 and 66+). Time since amputation was evaluated by dividing the sample into those who have been amputated for less than 1 year and those who had been amputated for 1 year or more. This cutoff was used as prosthesis users’ perception of their body image and their perception of different prosthetic designs were anticipated to differ in the first 12 months post amputation.

## Results

### Span of semantic space

The analysis of interview transcripts, together with results from focus groups, generated a total of 258 Kansei words (83 from transcripts and 175 from focus groups). Once duplicate words and versions of the same word (e.g. fun/funny) were removed 49 words remained. These were synthesised into 6 high-level Kansei words by the group of experts (Table 2). The final set of words were boring, functional, unique, cool, technological, and natural. The word “boring” was removed as it was agreed that this had negative connotations that were undesirable for prosthesis users. This left 5 high-level Kansei words which were considered to represent the span of semantic space of cosmetic covers for lower-limb prostheses.

**Table 2.** Kansei words describing prosthetic limbs, synthesised into categories represented by one high-level word.

Boring	Functional	Unique	Cool	Technological	Natural
Heavy	Athletic	Beautiful	Cool	Robotic	Human
Ugly	Flexible	Personal	Fun		Anatomical
Rustic	Strong	Modern	Cheeky	Futuristic	Authentic
Stiff	Sporty	Luxurious			Discrete
Dirty	Safe	Exclusive			Normal
Rigid	Practical	Designer		SciFi	Minimal
Formless	Adjustable	Enjoyable		Technical advanced	Traditional
Clumsy	Simple	Fashionable			Real
Hot	Moveable	Feminine			
Solid		Colourful			
		Nice			
		Different			
		Elegant			
		Sophisticated			

### Space of product properties

During a focus group discussion experts generated a list of product attributes that could be modified in a lower-limb prosthetic cosmetic cover to influence users’ Kansei. From this list they reached consensus on four product features (and their variations) that should be prioritised in this study; colour (neutral/colourful), form (organic/technical), surface (shiny/matt) and material (soft/hard).

### Synthesis - respondents

Table 3 presents respondent demographics. 53 lower-limb prosthesis users completed the questionnaire (26 female, 49%; 27 male, 51%). 29 competed the questionnaire while visiting prosthetic and orthotic clinics while 24 completed the questionnaire via the link posted in the closed Facebook forum. Most respondents were amputated below the knee (trans-tibial) (*n* = 39; 73.6%), due to trauma (*n* = 19; 35.8%). Time since amputation ranged from between 0 and 5 months (*n* = 12; 22.6%) to more than 10 years (*n* = 13;18.9%). The average score for participants level of satisfaction with their current prosthesis was 6.0 (SD = 3.0). 39 participants completed all items on the questionnaire (15 female, 24 male), in all cases of missing items participants had failed to rate one or more Kansei words (*n* = 16). In 2 cases participants had also failed to complete all items on the Big 5 inventory (extraversion sub-scale).

**Table 3.** Respondent demographics.

	*n*	%
Sex		
Male	27	51
Female	26	49
Age		
18-25	5	9.4
26-35	5	9.4
36-45	5	9.4
46-55	11	20.8
56-65	13	24.5
66+	14	26.4
Time since amputation		
0-5 months	12	22.6
6-11 months	8	15.1
1-3 years	12	24.5
4-10 years	8	15.1
10+ years	13	18.9
Amputation level		
Ankle	1	1.9
Trans-tibial	39	73.6
Knee disarticulation	2	3.8
Trans-femoral	10	18.9
Hip disarticulation	1	1.9
Cause of amputation		
Congenital	3	5.7
Trauma	19	35.8
Infection	1	1.9
Cancer	3	5.7
Vascular	5	9.4
Diabetes	10	18.9
Other	12	22.6
Current cosmetic cover design		
No cover only pipe	33	62.3
Shaped foam with nylon stocking	4	7.5
Shaped foam with silicon surface	5	9.4
Shaped hard shell	4	7.5
Special designed shell	3	5.7
Other	4	7.5

### Rating of Kansei words

**Figure 2.**
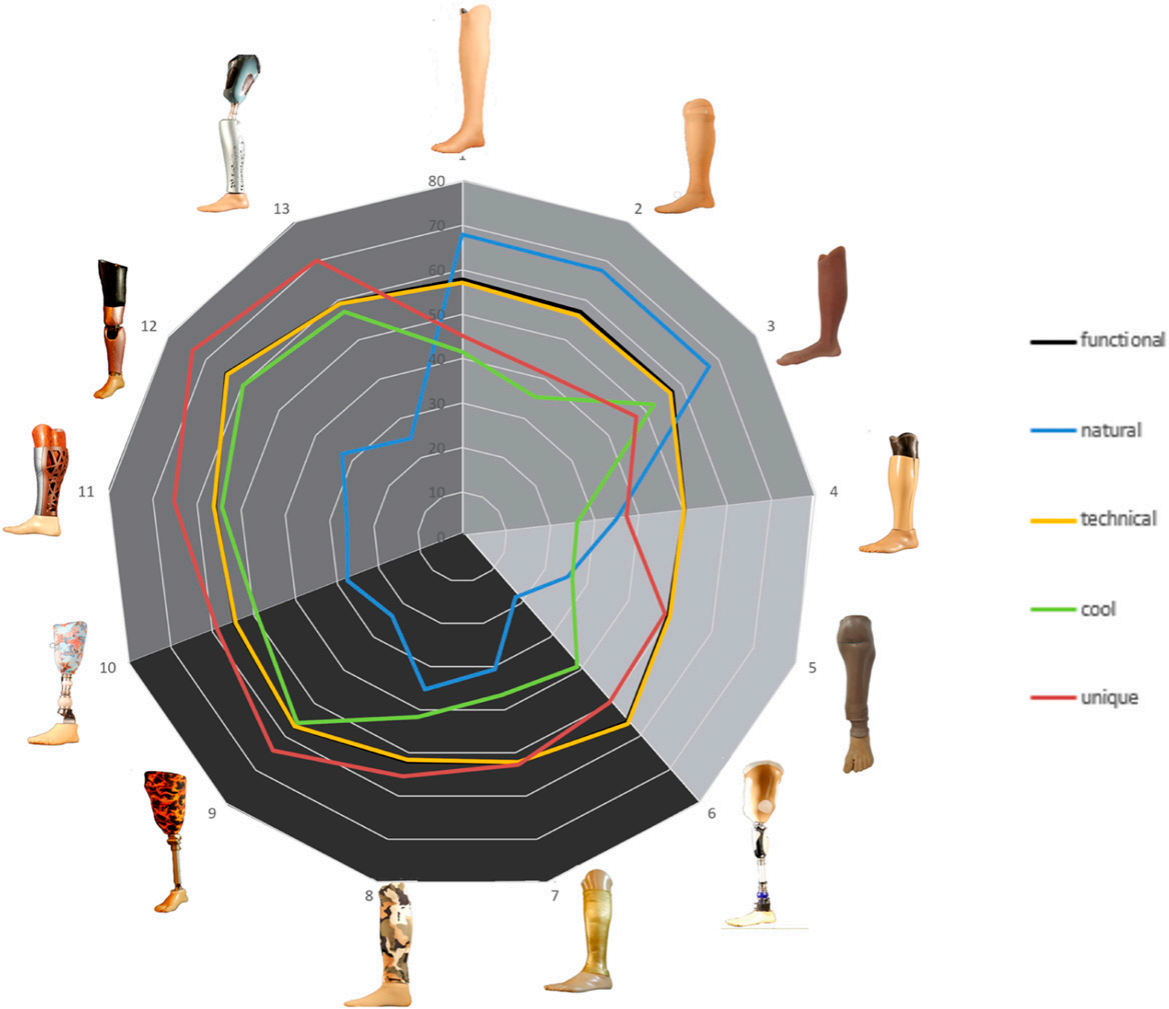
Rating of Kansei words for each prosthetic design. Shaded areas reflect categories of prosthesis design, realistic, functional and expressive. Different shading has been used to distinguish between expressive prostheses incorporating a purpose designed sock and expressive prosthesis made using a 3d printed shell.

Rating of Kansei words for all who responded to relevant items in the questionnaire is presented as a radar chart in Figure 2. Charts stratified by sex, age, time since amputation and extroversion subscales are presented in supplemental file A.

Visual inspection of Q-Q plots showed a symmetric distribution of data following a line representing the theoretical normal. Mean ratings for each Kansei word were subsequently compared across aesthetic design categories. Results are presented in Table 4 and indicate that there were significant differences across categories of prosthesis design for the Kansei words ‘Natural’, ‘Technical’, ‘Cool’ and ‘Unique’. For the Kansei word ‘Natural’, post hoc analysis revealed that prostheses categorised as realistic scored significantly higher than functional and expressive designs (*p* < 0.001). For the Kansei words ‘Technical’, ‘Cool’ and ‘Unique’ prostheses classified as expressive, and were 3d printed, scored significantly higher than all other designs (*p* < 0.001).

**Table 4.** Mean VAS ratings (standard deviation) of Kansei words for each category of prosthesis design (*n* = 37).

Kansei word	Prosthesis design				Comparison of means
	Realistic design	Functional design	Expressive design (sock)	Expressive design (3D printed)	
Functional	57.88 (27.31)	52.76 (26.91)	54.13 (22.98)	59.97 (21.09)	F (2.7100.1) = 2.65, *p* = 0.59
Natural	59.94* (27.16)	21.88 (20.58)	28.44 (22.79)	26.46 (20.03)	F (2.3, 86.0) = 45.67, *p* < 0.001
Technical	40.70 (26.19)	44.18 (27.47)	46.32 (27.36)	59.81* (22.80)	F (2.3, 82.4) = 10.37, *p* < 0.001
Cool	38.67 (30.82)	33.43 (28.70)	44.49^#^ (29.26)	54.08* (31.70)	F (1.9, 70.4) = 9.27, *p* < 0.001
Unique	43.86^#^ (27.94)	49.69 (29.33)	57.47^#*¤*^ (26.72)	69.68* (20.67)	F (2.7, 97.4) = 22.28, *p* < 0.001

*Significantly different from all other categories of prosthesis (*p* < 0.05), #significantly different from functional prostheses (*p* < 0.05). significantly different from realistic prostheses (*p* < 0.05).

MCC results are presented in supplemental file B. Values for the Kansei word “functional” were less than the acceptable threshold value (0.6) in all but two models (people aged 65 years and over and people who had been amputated for less than 1 year). This word was subsequently removed from further analyses. MCCs for all other words were above the threshold value. PCC results (supplemental file C) indicated that the highest degree of affinity was achieved between the Kansei words ‘Unique’, ‘Cool’ and ‘Technological’ and the product attributes ‘Colour’, and ‘Material’.

QT1 category scores explain how different product properties affect the Kansei of each product and are presented in Table 5. One can see from Table 5 that a prosthesis is perceived to be most ‘Unique’ when it is colourful and hard while a prosthesis is perceived to be ‘Natural’ when it is has a neutral colour, a matt finish and has a soft surface. Results also indicate that a prosthesis is most likely to be perceived as being ‘Unique’, ‘Cool’ and ‘Technological’ if it has a colourful as opposed to a neutral tone. The strength of this relationship was observed to be much greater for women compared to men and for people who had been amputated for 1 year or longer.

**Table 5.** QT1 category scores. Darker colours reflect a stronger positive (green) or negative (red) association.

Product feature	Product variations	Score Total	Female	Male	18-45	46-55	65+	<1 year	1 year +	Low	moderate	high
Unique												
Surface	Shiny	0.22	0.04	0.34	−4.84	1.18	2.02	0.71	−0.09	−1.41	0.25	1.58
	Matt	−0.26	−0.05	−0.39	5.65	−1.37	−2.36	−0.84	0.1	1.64	−0.29	−1.84
Colour	Colourful	7.86	11.05	5.86	6.41	9.17	6.92	3.18	10.78	6.61	8.36	7.44
	Neutral	−9.17	−12.89	−6.83	−7.48	−10.7	−8.08	−3.71	−12.58	−7.71	−9.75	−8.68
Material	Hard	3.05	4.56	2.11	6.85	2.85	0.99	0.92	4.39	2.66	3.03	3.46
	Soft	−6.87	−10.26	−4.75	−15.42	−6.4	−2.24	−2.06	−9.87	−5.98	−6.82	−7.78
Form	Technical	−3.06	−4.8	−1.97	−11.35	−3.86	3.15	−1.36	−4.12	−5.26	−1.87	−4.7
	Organic	0.92	1.44	0.59	3.4	1.16	−0.94	0.41	1.24	1.58	0.56	1.41
Cool												
Surface	Shiny	−2.83	−3.01	−2.71	−4.11	−1.83	−3.4	−2.72	−2.9	−3.57	−2.94	−1.84
	Matt	3.3	3.51	3.16	4.8	2.14	3.97	3.17	3.38	4.16	3.43	2.14
Colour	Colourful	5.56	7.91	4.1	3.24	9.1	2.1	2.58	7.43	6.86	4.72	6.95
	Neutral	−6.49	−9.22	−4.78	−3.78	−10.61	−2.44	−3.01	−8.66	−8.01	−5.51	−8.11
Material	Hard	2.71	4.69	1.47	6.32	3.37	−0.43	−0.43	4.67	1.87	3.11	2.23
	Soft	−6.09	−10.54	−3.31	−14.22	−7.58	0.97	0.97	−10.5	−4.21	−7	−5.01
Form	Technical	0.26	−1.65	1.45	−7.13	−4.41	11.28	3.32	−1.66	4.74	0.85	−5.44
	Organic	−0.08	0.5	−0.44	2.14	1.32	−3.39	−0.99	0.5	−1.42	−0.26	1.63
Technological												
Surface	Shiny	0.8	−1.5	2.23	−3.12	0.62	3.44	0.13	1.21	0.91	1	0.07
	Matt	−0.93	1.75	−2.6	3.64	−0.73	−4.01	−0.15	−1.42	−1.06	−1.17	−0.09
Colour	Colourful	4.88	5.16	4.7	3.69	7.73	1.65	4.21	5.3	5.56	4.03	6.82
	Neutral	−5.69	−6.02	−5.48	−4.31	−9.02	−1.93	−4.91	−6.18	−6.49	−4.7	−7.96
Material	Hard	2.41	2.74	2.21	3.27	3.56	0.31	1.93	2.72	2.92	2.08	2.99
	Soft	−5.43	−6.16	−4.98	−7.36	−8	−0.7	−4.33	−6.12	−6.58	−4.67	−6.73
Form	Technical	0.05	−2.89	1.89	−4.71	0.64	2.17	3.9	−2.35	1.84	0.79	−3.72
	Organic	−0.02	0.87	−0.57	1.41	−0.19	−0.65	−1.17	0.7	−0.55	−0.24	1.12
Natural												
Surface	Shiny	−7.16	−9.35	−5.79	−10.77	−6.74	−5.53	−6.49	−7.58	−7.71	−7.94	−4.33
	Matt	8.36	10.91	6.76	12.57	7.86	6.45	7.57	8.85	9	9.27	5.05
Colour	Colourful	−7.98	−6.64	−8.82	−9.25	−3.24	−13.76	−11.07	−6.05	−9.84	−8.96	−3.41
	Neutral	9.31	7.75	10.29	10.8	3.78	16.05	12.92	7.06	11.47	10.46	3.98
Material	Hard	−2.51	−0.85	−3.55	−0.66	−1.9	−4.51	−4.78	−1.1	−3.14	−2.64	−1.58
	Soft	5.66	1.92	7.99	1.48	4.27	10.15	10.75	2.48	7.07	5.94	3.56
Form	Technical	−7.37	−8.43	−6.71	−7.22	−9.33	−4.75	−11.86	−4.57	−2.91	−7.13	−11.99
	Organic	2.21	2.53	2.01	2.17	2.8	1.43	3.56	1.37	0.87	2.14	3.6

The Kansei word ‘Cool’ was positively associated with a matt surface on the prosthesis but this was generally not the case for the Kansei words ‘Unique’ and ‘Technological’. Prostheses perceived as being ‘Natural’ had a strong association with neutral colours. While a strong positive relationship between the word ‘Natural’ and prostheses with neutral colours was observed for all subcategories, the relationship was extremely high for people aged 65 years and over and for people who had been amputated for less than 1 year. A matt finish on prostheses was also positively associated with a ‘Natural’ Kansei, particularly for women and people aged between 18 and 45. Prostheses perceived as having a technical appearance (e.g. designs with no cosmetic cover) were negatively associated with the Kansei word ‘Natural’.

## Discussion

This study used a novel method, Kansei engineering, to increase our understanding of clients’ emotional responses to the cosmetic appearance of lower limb prostheses. The method enabled us to quantify the association between Kansei words representing the emotional responses elicited from prostheses with different aesthetic designs against specific product properties. Results of the study can be used in the clinical setting to support prosthesis users in their choice of cosmetic cover but can also be used by developers and designers of prosthetic componentry.

The high-level words identified in this study (‘Unique’, ‘Cool’, ‘Natural’ and ‘Technological’ and ‘Functional’) are not usual in the context of Kansei Engineering. Each of the words identified by our expert group have been previously used for Kansei Engineering applications. For example the word ‘Cool’ has been used in studies investigating the affective coherence of chocolate products,^17,25^ sunglasses^34^ and office chairs.^35^ The word ‘unique’ has been used in studies of food packaging,^36^ baby bags^37^ and USB flash drives. The word ‘Natural’ has been used in a study of computer interface design,^38^ the word ‘Technological’ in a study of USB flash drives^39^ and the word ‘Functional’ has been used the design of urban spaces^40^ and desks.^41^

In our study four of the identified Kansei words were found to have strong associations with product properties related to limb prostheses, these were ‘Unique’, ‘Cool’, ‘Natural’ and ‘Technological’. The Kansei word ‘Functional’ was not found to be a reliable predictor of product properties, likely due to difficulties in distinguishing between a device with a functional appearance and physical functionality of the prosthesis. Vlachaki et al.^12^ have previously used the term functional as a classification for prostheses which have a robotic design and no cosmetic cover. Based upon our results we argue that this is not an appropriate term for classifying these types of design. Unfortunately, no single Kansei word tested in this study was able to clearly distinguish prosthetic designs with no cosmetic cover from other designs and we suggest that more Kansei words should be tested before a classification system is accepted for general use.

The Kansei word ‘Natural’ had the strongest association with prosthetic design categories that Vlachaki et al.^12^ have classified under the heading of realistic prostheses (i.e. designs which mimic the look of a human limb). The product properties that were most strongly associated with the Kansei word ‘Natural’ were neutral colours and a matt finish. In contrast, the Kansei words ‘Unique’, ‘Cool’ and ‘Technological’, were most strongly associated with expressive prostheses^12^ that comprised of hard 3d printed shells and had a colourful appearance. These results are of relevance for clinical prosthetists who should assist users in narrowing down design choices related to the aesthetic appearance of their prosthetic device. For example, a client who wishes to have a prosthesis that elicits a ‘Natural’ feeling should be offered choices from the range of realistic prosthetic designs that incorporate a matt finish, while clients who express a desire for a ‘Unique’ prosthesis should be offered choices from a range of expressive prostheses and will most likely prefer a 3d printed shell with a colourful appearance. Designers of prosthetic components can also benefit from these results by using them to guide design decisions. For example, a person designing a cosmetic cover with a ‘Natural’ Kansai should incorporate neutral colours and a matt finish into their product.

Personal characteristics of prosthesis users appeared to affect the strength of relationships between of Kansei words and product properties however the direction of ratings were generally consistent. One exception can be found in ratings of the Kansei word ‘Cool’ by participants who were over the age of 65. This group rated prostheses which had a technical rather than organic form as having a strong positive association with the word ‘Cool’ while groups aged under 65 rated these prostheses as having a negative association. This suggests that people over the age of 65 have different view on what they consider as cool when compared to younger age groups and is a good reminder for clinicians and designers that they should not to make assumptions based upon their own perceptions of Kansei words.

Previous work has reported that design preferences for prosthetic cosmeses differs between males and females, although results to date present conflicting findings.^12–14^ While the current study did not specifically investigate design preferences, our results show that designs are perceived in a similar way by males and females. It is subsequently unlikely that differences in design preferences are due to differences in the emotional response of males versus females to specific prosthetic design characteristics. Females did however tend to be more extreme than their male counterparts when rating Kansei words, using a wider span of the rating scale. The extent to which this may affect design preferences could not be determined in this study.

Personality traits have long been associated with consumers’ choice of products^42^ and there has been some suggestion that personality traits may be related to the preference for realistic versus expressive prostheses.^10,11^ In this study we demonstrate that the perceptions of different prosthetic designs between people classified as having low, moderate, or high extroversion are quite similar. This indicates that the association between product properties and Kansei words is unlikely to be influenced by personality types and that the Kansei words can be useful to help people representing a range of personality types to express their desire for prosthetic design.

Design choices available to prosthesis users have grown exponentially over the past decade, largely thanks to companies offering 3d printed covers and the option for users to select from a wide range of designs, colours and textures.^43^ While availability of choice should be welcomed, care should be taken to ensure that the abundance of options available do not overwhelm prosthesis users. This phenomenon is well understood by marketing professionals and is recognised as “the paradox” of choice,^44^ proposing that, while offering some choice works to empower consumers, an abundance of options can be perceived as overwhelming, creating anxiety and stress. We propose that use of Kansei words identified in this, and future studies can be used to identify the feeling clients’ desire from a prosthesis and help to narrow the abundance of options from which they need to choose.

### Limitations

The accuracy of the Kansei Engineering method is affected by the suitability of Kansei words that are chosen to be included in the evaluation.^35^ While this study used two well accepted methods for identifying appropriate words, the hierarchization of words by our expert group was ultimately subjective. Several different statistical methods to gather, select and hierarchize Kansei words have been proposed.^35,45^ Use of these methods when selecting Kansei words may have improved the accuracy of our results.

No criterion currently exists to determine the appropriate sample size for Kansei engineering studies and size varies greatly in the current literature.^27^ Marco-Almagro^27^ report that 30% of reviewed Kansei engineering studies were conducted with fewer than 24 subjects while 10% were conducted with more than 80 subjects. Despite the lack of clear guidelines, it is acknowledged that the sample size of this study may have had an impact on the results, particularly when stratified into sub-groups.

To quantify the relationship between Kansei words and product properties we chose to use Hayashi’s QT1. While this is recognised as an effective analysis method^46^ and is the most widely used tool for synthesising data in Kansei engineering studies, the method does not account for the variability among individuals. To address this issue some authors have recommended that alternate methods which may be more appropriate.^27^ Ordinal Logistic Regression is one such approach.

Previous work has demonstrated that cultural context is an important factor in the aesthetic design of prostheses^7^ and that many products (e.g. cars, coffee, stereo systems) are perceived differently by individualist rather than collectivist cultures.^47^ This study was conducted in Sweden, a highly individualistic culture^48^ in which inhabitants would be more likely to seek products that reflect their person style. While results can be useful for design of prostheses in the Swedish context it will be necessary to replicate the study in other countries before generalising the results to other contexts.

## Conclusion

In this paper explored if Kansei Engineering can help us to understand the relationship between emotional responses of users and aesthetic design features in prosthetic limbs. The approach was successfully applied, and we managed to established links between four Kansei words and specific aesthetic design features. Strong positive associations were observed between the Kansei word ‘Natural’ and design characteristics associated with realistic prostheses that mimic the look of a human extremity. The Kansei words ‘Unique, ‘Technological’ and ‘Cool’ were all positively associated with expressive prosthetic designs with hard shells and colourful appearances. Few differences were observed when comparing ratings of Kansei words between sub-groups within the sample (e.g. male/female; introvert/extrovert). In future work it will be important to determine how user characteristics influence aesthetic design preferences and to explore if there are additional Kansei words that may be useful to support prosthesis users in their design choices.

## Supplemental Material


Supplemental Material - Designing feelings into lower-limb prostheses – A kansei engineering approach to understand lower-limb prosthetic cosmese
Supplemental Material for Designing feelings into lower-limb prostheses – A kansei engineering approach to understand lower-limb prosthetic cosmese by Nerrolyn Ramstrand, Maria Riveiro, Lars Eriksson and Michael Ceder in Journal of Rehabilitation and Assistive Technologies Engineering.
