# Supplementary material for: Designing feelings into lower-limb prostheses – A kansei engineering approach to understand lower-limb prosthetic cosmeses
Source: J Rehabil Assist Technol Eng. 2024 Oct 17;11:20556683241289938. doi: 10.1177/20556683241289938 (PMC11489910; doi:10.1177/20556683241289938)
Supplement: Supplemental Material - Designing feelings into lower-limb prostheses – A kansei engineering approach to understand lower-limb prosthetic cosmese [file sj-pdf-1-jrt-10.1177_20556683241289938.pdf]

Supplementary file A:

Rating of Kansei words stratified by sex, age, time since amputation and extroversion scores.

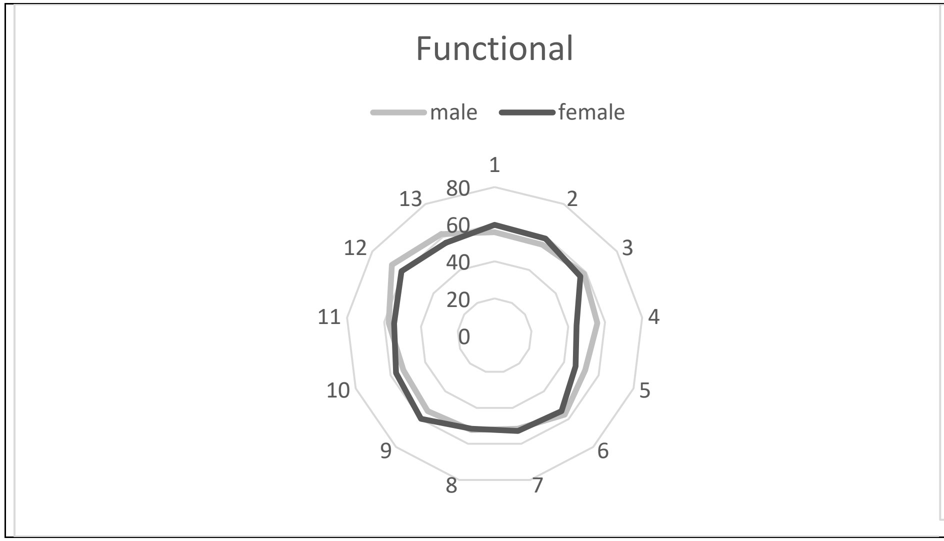

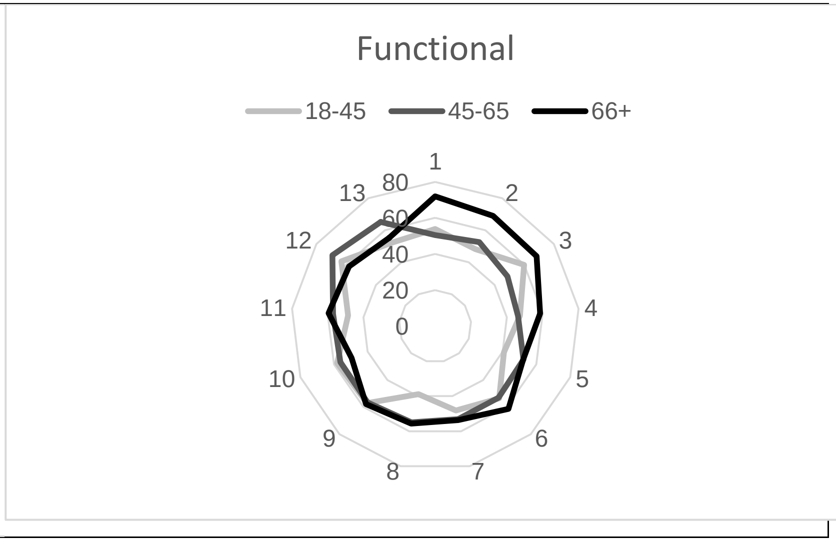

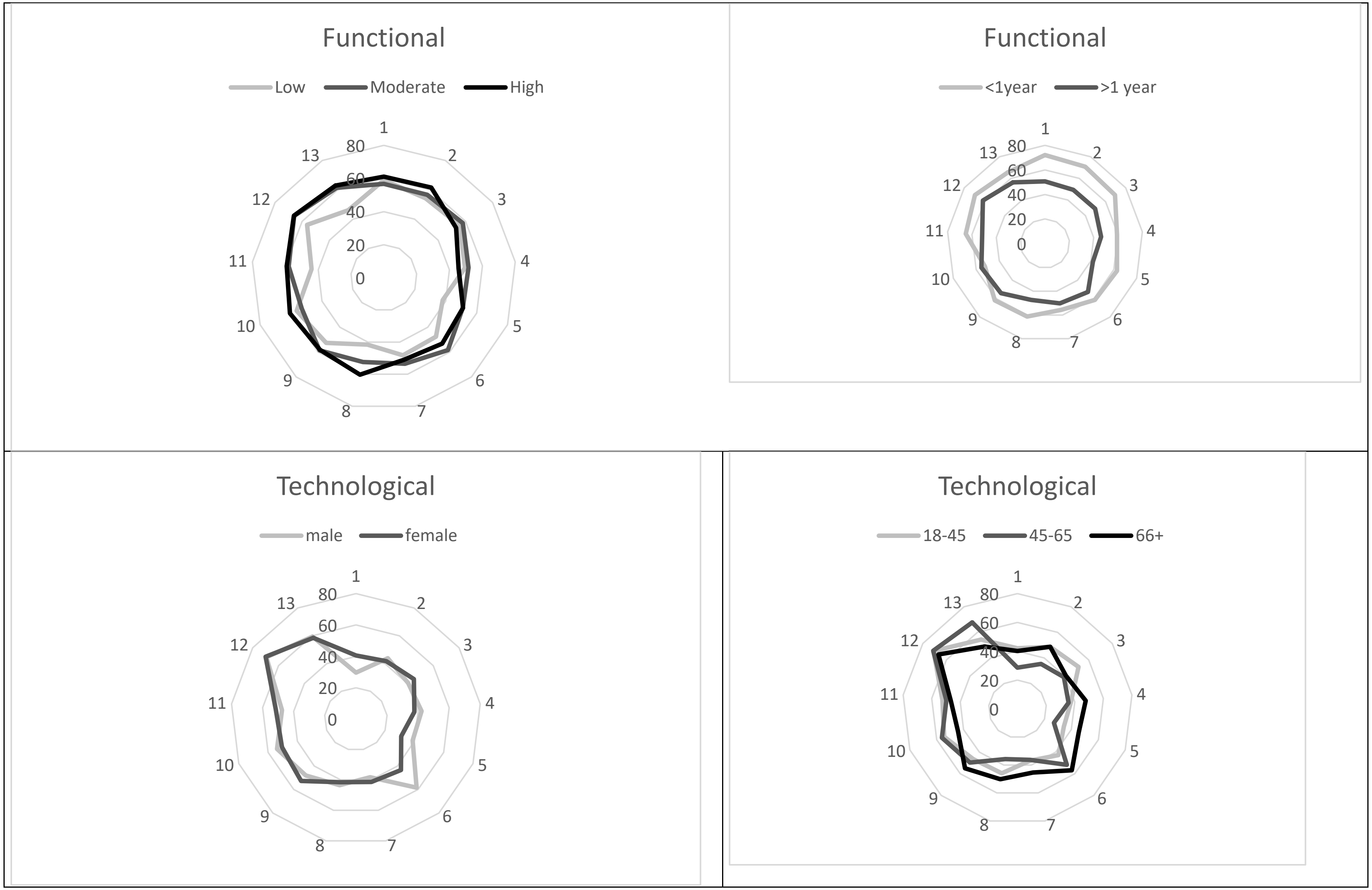
Functional
Low
Moderate
High
1
2
3
4
5
6
7
8
9
10
11
12
13
80
60
40
20
0
Functional
<1year
>1 year
1
2
3
4
5
6
7
8
9
10
11
12
13
80
60
40
20
0
Technological
male
female
1
2
3
4
5
6
7
8
9
10
11
12
13
80
60
40
20
0
Technological
18-45
45-65
66+
1
2
3
4
5
6
7
8
9
10
11
12
13
80
60
40
20
0

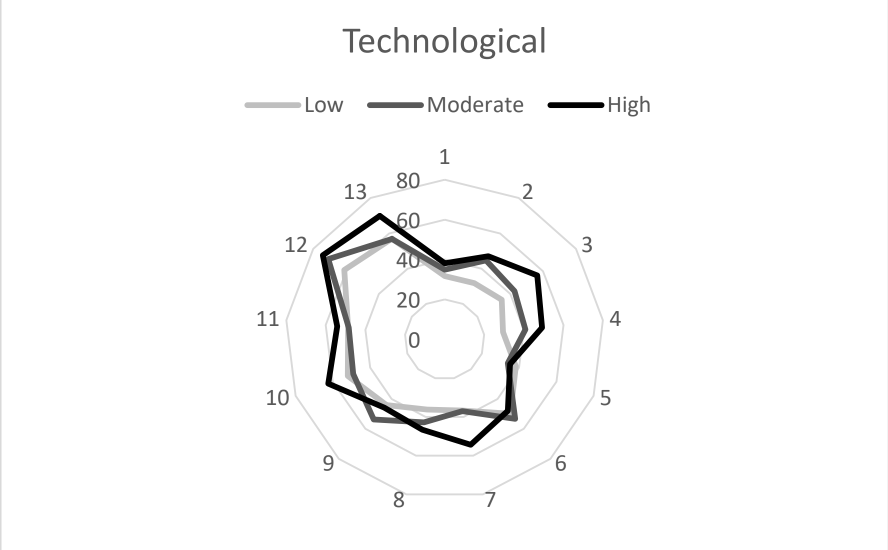
Technological
Low
Moderate
High
1
2
3
4
5
6
7
8
9
10
11
12
13
0
20
40
60
80

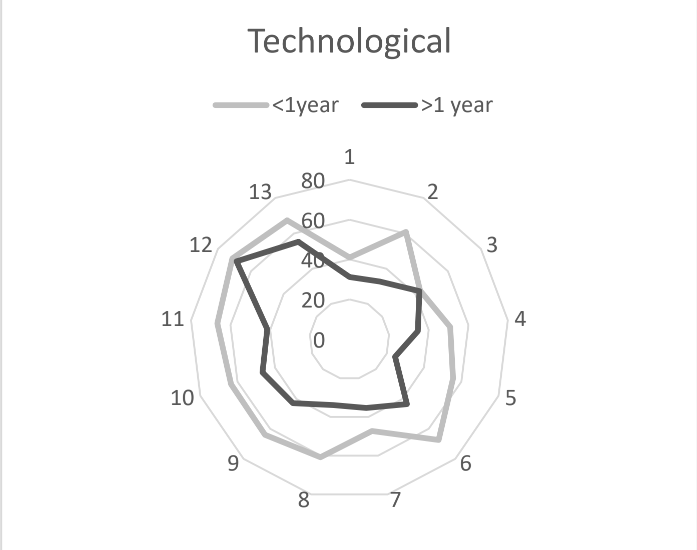
Technological
<1year
>1 year
1
2
3
4
5
6
7
8
9
10
11
12
13
0
20
40
60
80

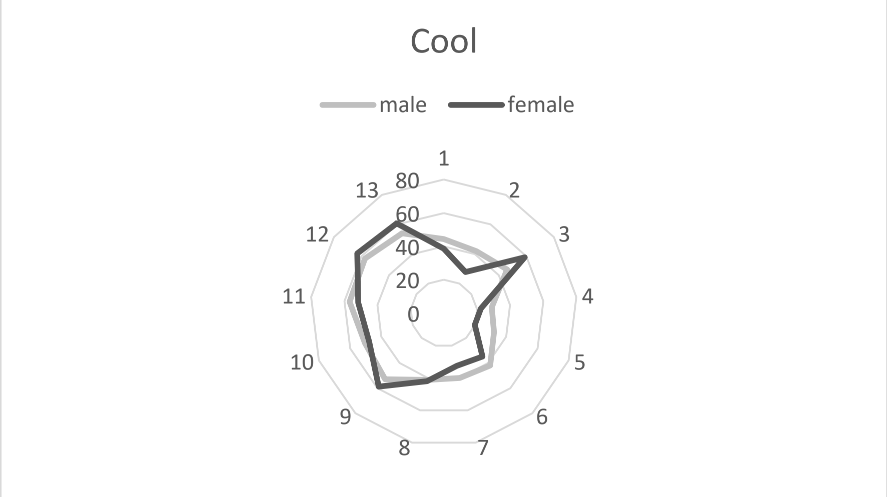
Cool
male
female
1
2
3
4
5
6
7
8
9
10
11
12
13
0
20
40
60
80

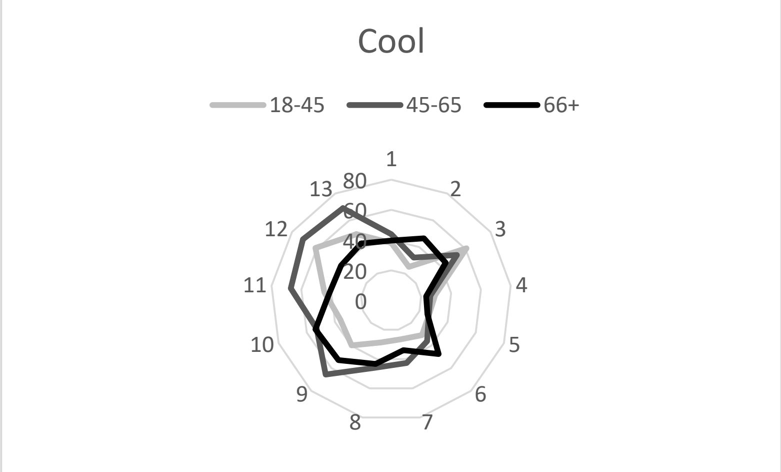
Cool
18-45
45-65
66+
1
2
3
4
5
6
7
8
9
10
11
12
13
0
20
40
60
80

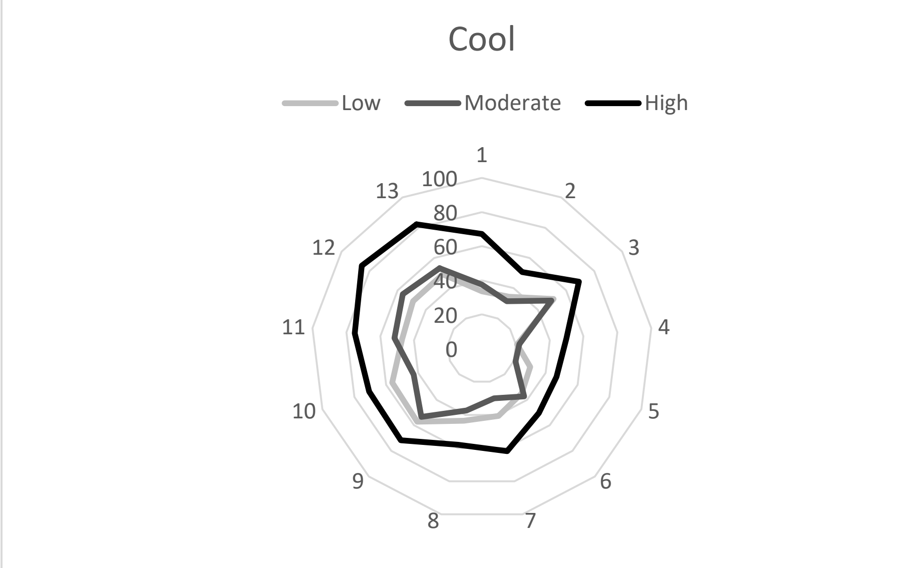
Cool
Low
Moderate
High
1
2
3
4
5
6
7
8
9
10
11
12
13
100
80
60
40
20
0

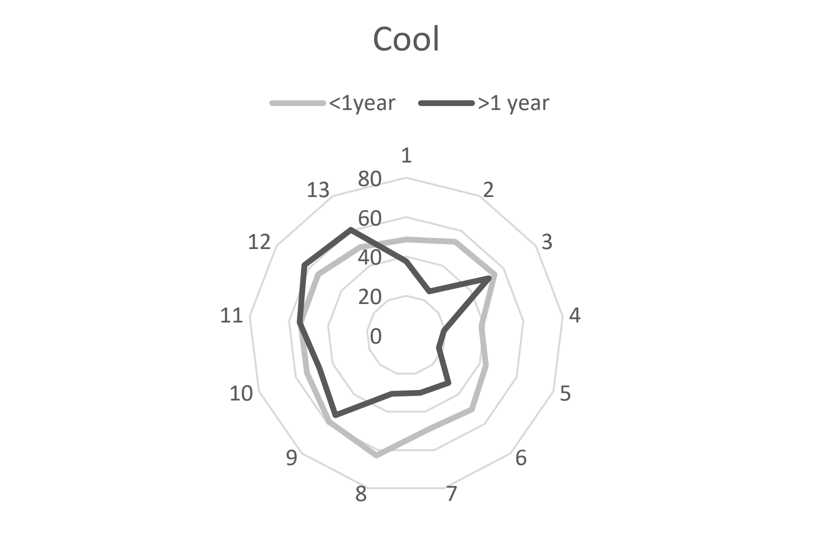
Cool
<1year
>1 year
1
2
3
4
5
6
7
8
9
10
11
12
13
80
60
40
20
0

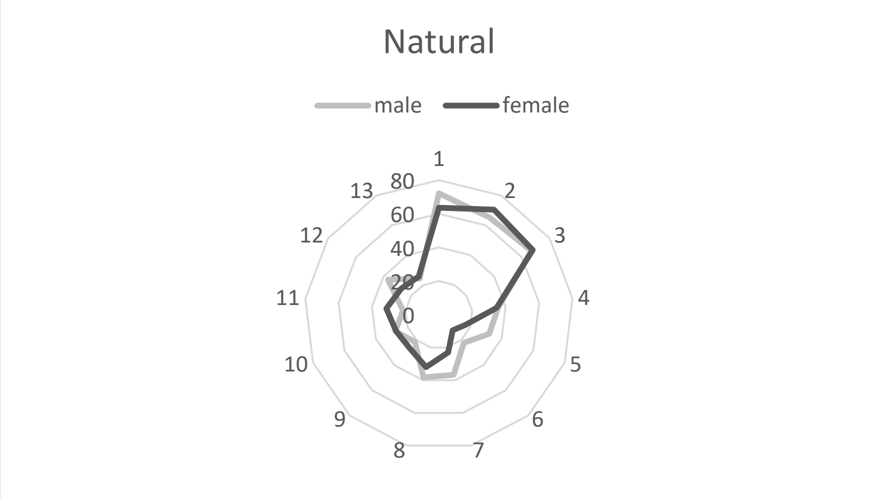
Natural
male
female
1
2
3
4
5
6
7
8
9
10
11
12
13
80
60
40
20
0

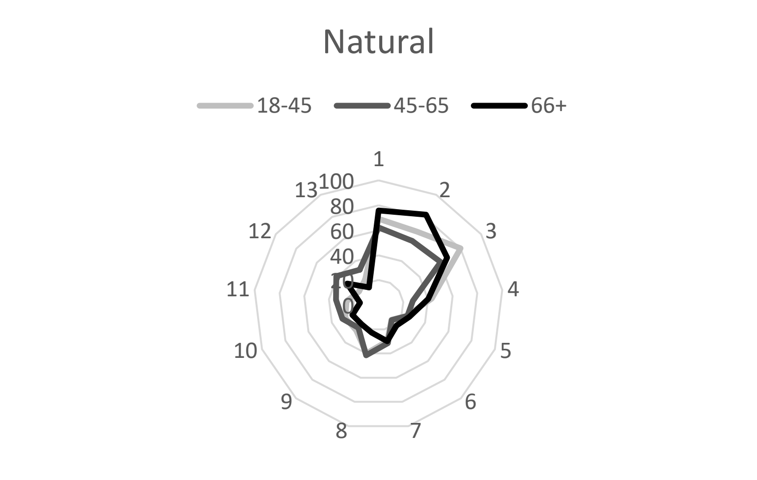
Natural
18-45
45-65
66+
1
2
3
4
5
6
7
8
9
10
11
12
13
100
80
60
40

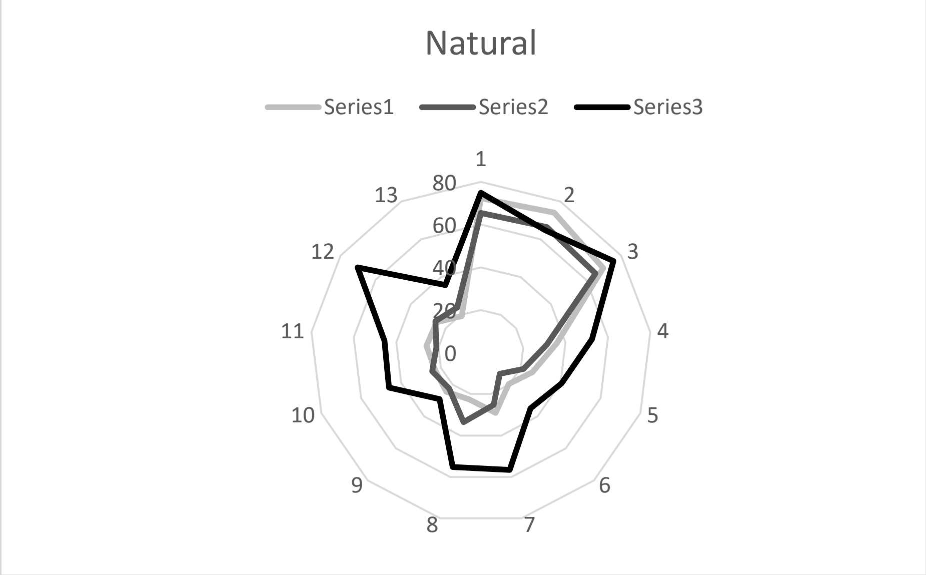
Natural
Series1
Series2
Series3
0
20
40
60
80
1
2
3
4
5
6
7
8
9
10
11
12
13

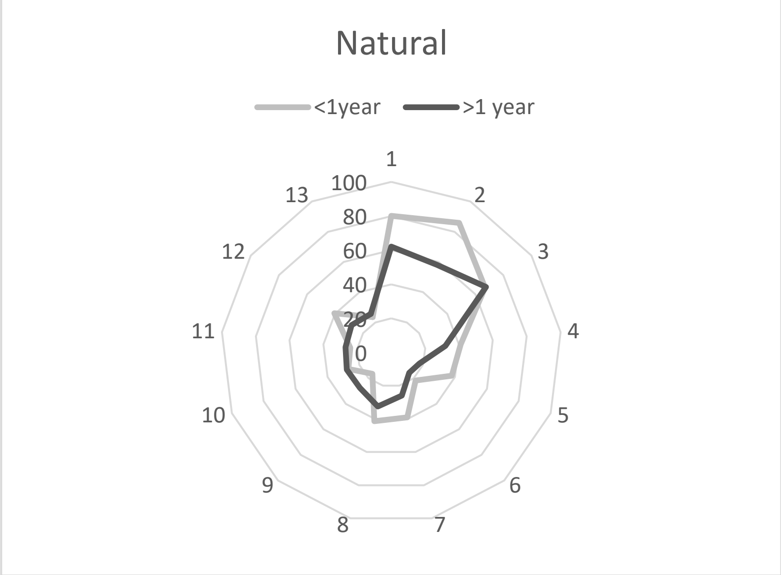
Natural
<1year
>1 year
0
20
40
60
80
100
1
2
3
4
5
6
7
8
9
10
11
12
13

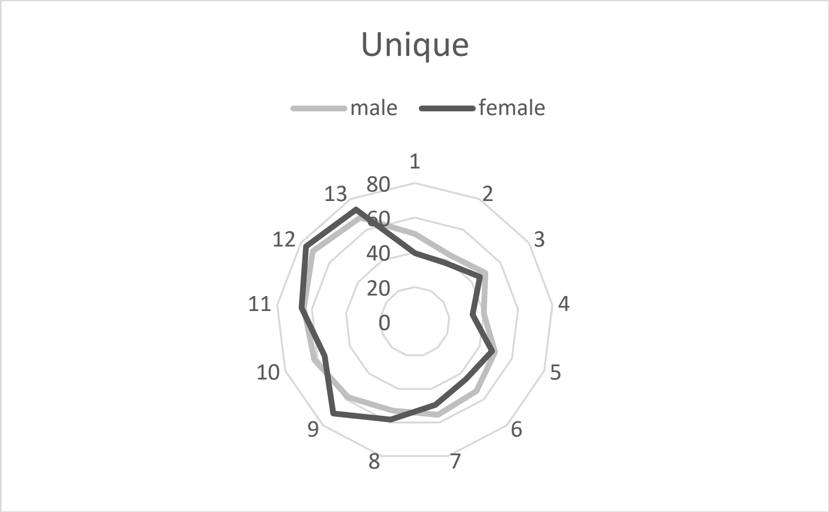
Unique
male
female
0
20
40
60
80
1
2
3
4
5
6
7
8
9
10
11
12
13

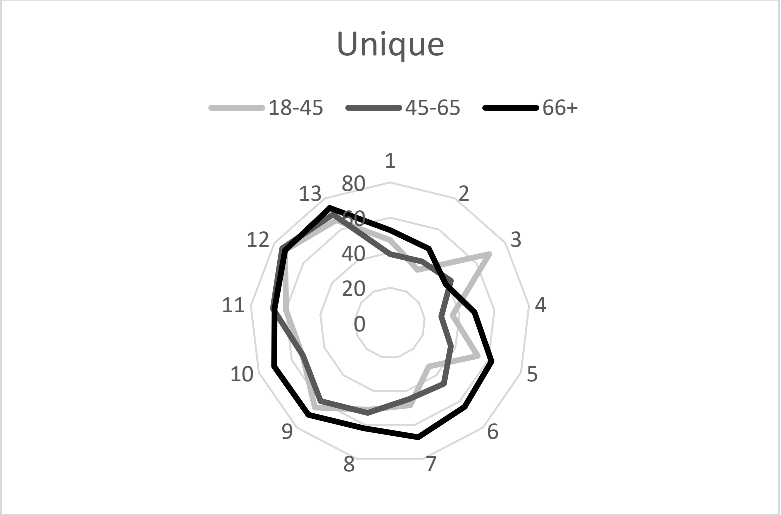
Unique
18-45
45-65
66+
0
20
40
80
1
2
3
4
5
6
7
8
9
10
11
12
13

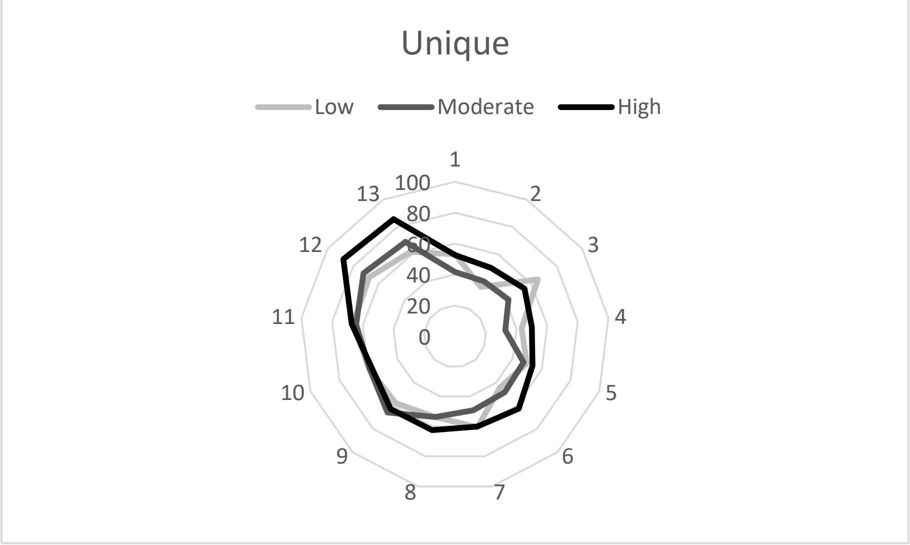

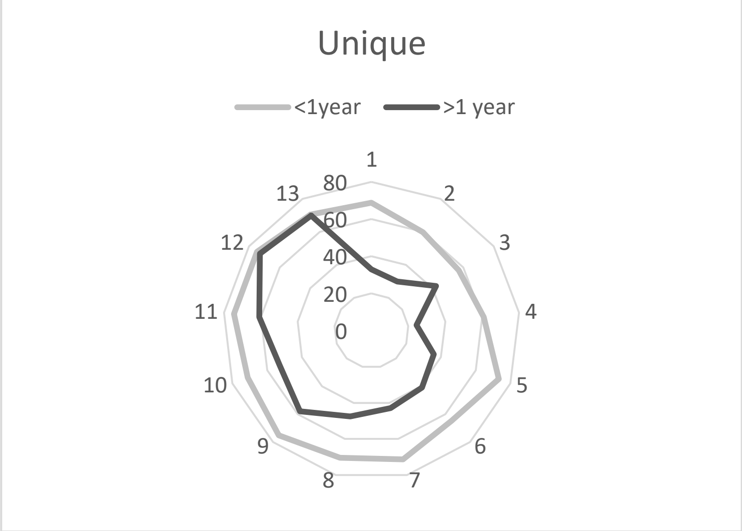

Supplementary file B

	Product feature	Score Total	Female	Male	18-45	46-55	65+	<1 year	1 year +	Low	moderate	high
MCC	Unique	0,91	0,91	0,87	0,78	0,88	0,90	0,79	0,91	0,85	0,89	0,87
	Cool	0,76	0.74	0,79	0,72	0,81	0,84	0,77	0,77	0,87	0,71	0,79
	Technological	0,71	0.64	0,75	0,53	0,76	0,67	0,71	0,68	0,79	0,62	0,70
	Natural	0,85	0.81	0,86	0,82	0,85	0,84	0,90	0,80	0,79	0,86	0,71

	Functional	0.18#	0,36#	0,50#	0,44#	0,56#	0,74	0,67	0,35#	0,46#	0,20#	0,62

Multiple correlation coefficients (MCC) reflecting goodness of fit of the linear regression model for each Kansei word and each sub-category of interest. *#=MCC score below acceptable threshold value (0,6)*

Supplementary file C

		Total	Female	Male	18-45	46-55	65+	Amp <1 year	Amp 1+ years	Low	Moder ate	High
Unique	Surface	0,21	0,19	0,21	0,08	0,26	0,29	0,23	-0,17	-0,12	0,21	0,26
	Colour	0,69	0,8	0,62	0,62	0,74	0,64	0,5	0,82	0,64	0,72	0,64
	Material	0,61	0,69	0,55	0,7	0,6	0,5	0,49	0,7	0,58	0,61	0,59
	Form	0,14	0,21	0,1	0,33	0,17	0,03	0,08	0,21	0,19	0,11	0,16
Cool	Surface	-0,01	0,05	-0,04	0,13	-0,07	0,01	-0,09	0,08	0,07	0,04	-0,12
	Colour	0,64	0,69	0,58	0,53	0,72	0,5	0,49	0,71	0,7	0,62	0,6
	Material	0,59	0,65	0,53	0,73	0,61	-0,42	-0,44	0,68	0,56	0,62	0,54
	Form	-0,04	0,1	-0,01	0,28	0,18	0,28	0,02	0,11	0,12	-0,02	0,16
Technological	Surface	0,23	-0,1	0,31	-0,02	0,2	0,36	0,2	0,25	0,24	0,24	0,19
	Colour	0,6	0,6	0,6	0,54	0,69	0,47	0,54	0,63	0,67	0,56	0,62
	Material	0,57	0,57	0,56	0,59	0,61	0,46	0,53	0,58	0,62	0,54	0,57
	Form	-0,05	0,13	0,01	0,18	-0,02	0,01	0,04	0,13	0,03	-0,02	0,14
Natural	Surface	0,07	0,17	0,01	0,18	0,08	-0,02	-0,01	0,13	0,08	0,11	-0,05
	Colour	-0,08	-0,11	-0,06	-0,04	-0,26	0,1	-0,05	-0,11	-0,01	-0,02	-0,28
	Material	-0,33	-0,39	-0,3	-0,4	-0,36	-0,26	-0,28	-0,38	-0,3	-0,32	-0,38
	Form	0,18	0,21	0,17	0,17	0,24	0,12	0,22	0,15	0,1	0,18	0,24
Functional	Surface	-0,18	-0,16	-0,2	-0,09	0,23	-0,16	-0,15	0,21	-0,11	0,21	-0,16
	Colour	0,403	0,43	-0,38	0,42	0,48	-0,28	-0,34	0,46	-0,35	-0,39	0,47
	Material	0,467	-0,42	0,49	0,51	0,48	-0,43	0,46	0,47	-0,44	0,46	0,5

	Form	0,08	0	0,13	0,02	0,12	0,08	0,12	-0,03	0,03	0,08	0,14

Partial correlation coefficients (PCC) reflecting the strength of relationship between product properties and Kansei words for each subcategory of interest. Amp=amputation.
